# The Test–Retest Reliability of the Bruininks–Oseretsky Test of Motor Proficiency-Short Form in Youth with Down Syndrome—A Pilot Study

**DOI:** 10.3390/ijerph18105367

**Published:** 2021-05-18

**Authors:** Vincenzo G. Nocera, Aaron P. Wood, Angela J. Wozencroft, Dawn P. Coe

**Affiliations:** 1 Department Health and Human Performance, Plymouth State University, Plymouth, NH 03264, USA; 2 Department of Kinesiology, Recreation, and Sport Studies, University of Tennessee, Knoxville, TN 37996, USA; awood46@vols.utk.edu (A.P.W.); awozenc1@utk.edu (A.J.W.); dcoe@utk.edu (D.P.C.)

**Keywords:** motor skills, disability, adolescence, intellectual disability, BOT-2 SF

## Abstract

Background: It is unclear whether assessments of motor proficiency are reliable for individuals with Down syndrome. The purpose of the study was to evaluate the test–retest reliability of the Bruininks–Oseretsky Test of Motor Proficiency-Short Form (BOT-2 SF) in youth with Down syndrome. Methods: Ten youth (ages 13.1–20.7 years) with Down syndrome completed the BOT-2 SF (14 items) plus a standing long jump on two separate occasions. Intraclass correlation coefficients (ICC), 95% confidence intervals (CIs), and standard error of measurement (SEM) were calculated to determine the test–retest reliability of the BOT-2 SF and the standing long jump. Results: The test–retest reliability of the BOT-2 SF overall scores and percentile rankings were considered excellent. The test–retest reliability of each of the subtests varied with classifications of poor (*n* = 5), fair to good (*n* = 6), and excellent (*n* = 4). Conclusion: Current evidence suggests that children with Down syndrome have reduced motor skills. However, there appears to be a lack of assessment tools that reliably evaluate the motor skills of this population. The results from this investigation suggest that the BOT-2-SF provides “excellent reliability” (≥0.75) to assess the motor skills in youth with Down syndrome.

## 1. Introduction

Down syndrome is a condition that results from a genetic abnormality at chromosome 21 [1] and is considered the most common genetic cause of intellectual disability. Down syndrome impacts between 6.7 and 8.3 per 10,000 inhabitants in the United States [2,3]. Due to its unique phenotype, Down syndrome is associated with multiple congenital abnormalities [1]. Comorbidities of Down syndrome include respiratory, cardiovascular, sensory, gastrointestinal, hematological, immune, endocrine, musculoskeletal, renal, genitourinary, and neurological conditions [4]. Since Down syndrome impacts almost every organ system, there is a wide spectrum of consequences that differ from the phenotype of their peers [5].

Current evidence suggests that approximately 30% of youth with Down syndrome are classified as obese [6,7], which is higher than what is reported for youth without disabilities (17%) [8] and those with other causes of intellectual disabilities (12–30%) [9,10]. A recent study by Diaz (2020) found that children with Down syndrome (mean age 12.5 ± 2.9) are 45% less likely to participate in regular physical activity and 52% less likely to participate in recreational sports compared to youth without disabilities. Furthermore, youth with Down syndrome are more likely to engage in high volumes of television viewing (>2 h per day) [11]. As such, physical activity is recommended to prevent excessive weight gain and promote weight loss [1,11]. However, the physical activity levels of individuals with Down syndrome remain low, and the majority of youth with Down syndrome do not meet national recommendations of 60 min of daily moderate to vigorous physical activity [12,13,14]. Thus, researchers are tasked with developing strategies to increase the physical activity levels of individuals with Down syndrome.

The low physical activity levels of youth with Down syndrome may be partially explained by poor motor skills in this population, as current literature suggests a direct association between motor skills and physical activity [15]. It has been documented that youth with Down syndrome have poorer motor skills compared to their peers [16,17]. This is important, as children and adolescents who are proficient in gross motor skills tend to participate in more physical activity [18,19,20,21]. Conversely, poor motor skill levels, indicating low motor proficiency, are associated with anxiety, poor social behaviors [22], and a tendency to avoid play and sport participation [23].

A variety of assessment tools for motor development and proficiency are commonly used in youth with Down syndrome. These tools include the Test of Gross Motor Development (TGMD-2 and TGMD-3), the Motor Assessment Battery for Children (MABC-2), and the Bruininks–Oseretsky Test of Motor Proficiency (BOT-2). These assessments were not developed for youth with disabilities, and normative data are typically not available for this population. Researchers have established test–retest reliability and internal validity for the TGMD-2, TGMD-3, and the MABC-2 in youth with Down syndrome [24]. Typically, studies that have looked at test–retest reliability and internal validity in specific clinical populations, such as Down syndrome, have included a small sample size due to the low prevalence in the population outside of the clinical setting [25,26,27]. Furthermore, studies in youth with Down syndrome often require multiple visits that may take place in addition to other programs/clinical visits for the participants, creating a burden that further complicates the recruitment process. Although these studies involved small samples and had wide age ranges, there was merit to these studies, as they made important contributions to the literature and aided in identifying assessment tools that were valid and could be reliably used with special populations, such as Down syndrome.

The BOT-2 was designed to measure motor proficiency of youth ages 4 to 21 years, as well as to identify motor impairments. Moreover, it has been used in youth with and without disabilities [28,29,30,31]. Lam, Rubin, White, Duran, and Rose (2018) recently established the test–retest reliability for the BOT-2 Complete Form (BOT-2 CF) for youth with Prader–Willi Syndrome [31]. However, currently, there have not been any studies that have examined the test–retest reliability of the BOT-2 Short Form (BOT-2 SF) and specifically focused on youth with Down syndrome.

The BOT-2 SF has been frequently used as a motor skill proficiency assessment tool in a variety of settings including adapted physical education, occupational therapy, and physical therapy [32]. For example, Lee, Hong, and Park (2018) found that approximately 43% of occupational therapists used the BOT-2, making it the most commonly used test to assess motor proficiency [33]. The BOT-2 CF has been shown to have comparable test–retest reliability for both children with and without disabilities (α = 0.92 with disabilities; α = 0.95 without disabilities) [30,31]. Specifically, the BOT-2 CF has shown excellent test–retest reliability and internal consistency for children, ages 4 to 12, with intellectual disabilities [34]. Furthermore, Ruiz-González and colleagues (2019) found that the BOT was the one of the most commonly used assessment tools of motor skills in the research setting for youth with Down syndrome [35]. However, the BOT-2 CF involves 53 items and takes approximately 45–60 min to complete for a typically developing child. The length of this assessment may not be ideal for youth with Down syndrome to complete, as they may not be able to comprehend instructions for all of the different assessments and may lose interest and focus during the extended testing period. A more concise version, the BOT-2 SF, has been developed [30]. The BOT-2 SF includes assessments from each BOT-2 subtest (14 items) with a completion timeframe of 15–20 min. The BOT-2 SF is highly correlated with the BOT-2 CF (r = 0.80) and is considered a practical assessment for use in youth with intellectual and developmental disabilities, as it is ideal for youth with limited memory capacity [36]. However, the test–retest reliability of the BOT-2 SF for youth with Down syndrome remains unclear. Thus, the purpose of this investigation was to evaluate the test–retest reliability of the BOT-2 SF for measuring motor proficiency in youth with Down syndrome.

## 2. Methods

### 2.1. Participants

A total of 10 individuals with Down syndrome (5 females), ages 13.1 to 20.7 years old, volunteered to participate in the investigation. Three participants were in the early to middle adolescent age range (13–17 years old), and seven were classified as later adolescents (18–20 years old). Participants were recruited through social media, including groups specific to youth with Down syndrome, and by word of mouth. All participants came into the research laboratory for a total of two visits. During the first visit, a detailed description of the study protocol was provided to the participants as well as parents and/or caregivers. Subsequent to the explanation of the study, parents/caregivers provided informed consent and the participants provided written assent. The protocol for this investigation was reviewed and approved by the affiliated Institutional Review Board/Ethics Committee. The research in the current study was carried out following the rules of the Declaration of Helsinki. During this time, parents/caregivers were asked if their child had any atlantoaxial instability, which is prevalent in youth with Down syndrome, to determine if high impact jumping activities were contraindicated. Next, anthropometric assessments of each participant’s height and weight were completed. Parents/guardians reported the date of birth and race/ethnicity of their children. Finally, participants completed the BOT-2 SF and standing long jump test. During the second visit, the participants completed only the BOT-2 SF and standing long jump test. To account for any training effect, all visits were separated by at least a week. No intervention or other testing by the current researchers was implemented between the visits. The participants’ parents/guardians did not report any other intervention or testing during this time period, and participants were encouraged to maintain their typical activity behaviors. Prior to each assessment, a demonstration was performed by a trained researcher.

### 2.2. Bruininks–Oseretsky Test of Motor Proficiency, Second Edition (BOT-2 SF)

The BOT-2 SF is a revision of the Bruininks–Oseretsky Test of Motor Proficiency [37]. This test is designed to measure fine and gross motor skills of youth ages 4 to 21 years as well as to identify motor impairments [28]. To maintain consistency, the same trained researcher administered all tests. Prior to data collection, the researcher was extensively trained by an experienced evaluator on administration techniques, scoring, and interpretation using the BOT-2 manual, training video, and numerous practice rating trials until proficiency was attained. Participants had an opportunity for breaks in order to prevent possible fatigue and frustration. Prior to the BOT-2 SF assessment, hand and leg dominance were determined for the participants by throwing and kicking a ball (one time each) and was confirmed with writing and balancing on the beam during the assessment. It should also be noted that all participants used the same hand and leg during both visits, further confirming dominance. The length of time to administer the battery of tests varied, and all assessments lasted less than one hour in duration.

The BOT-2 SF consists of 14 items, which address all of the BOT-2 subtests to provide a total motor composite score. The subtests include *fine motor precision*, *fine motor integration*, *manual dexterity*, *body coordination*, *balance*, *running speed and agility*, *upper-limb coordination*, and *strength*. The assessments for each subtest can be found in Table 1. Due to potential hypotonia, all participants in this investigation performed knee push-ups. A detailed description of each of these assessments can be found in the BOT-2 Manual [30]. Raw scores from each of the subtests were converted into point scores. Once converted, point scores were summed to provide a total point score. Total point scores were converted to standard scores and percentile rankings based on sex, age, and type of push-up using Appendix B in the BOT-2 Manual [30].

### 2.3. Standing Long Jump

Due to concerns of hypotonia that can be present in individuals with Down syndrome, the standing long jump was also used to determine strength. Lam et al. (2018) found the standing long jump to have the highest test–retest reliability of all of the BOT-2 strength components in youth with Prader–Willi syndrome [31]. This assessment also provides a measure of lower body strength, while the other strength components of the BOT-2 SF (push-ups and sit-ups) focus on upper body and core strength. This would provide a more comprehensive assessment of total body strength. Before the test, a tape measure was affixed to the floor to measure jump length. Prior to testing, participants were instructed to stand close to the starting line and to use a counter movement jump, arms back and knees bent before take-off, to correctly complete the long jump. Participants were asked to remain in the landing spot following the jump. Jump distance was taken from the starting line to the posterior edge of the heel of the foot closest to the origin of the jump.

### 2.4. Statistical Analysis

Data were analyzed using SPSS version 25 (IBM, Armonk, NY, USA). Descriptive characteristics of the participants are presented as mean ± standard deviation (SD). All participants completed each assessment in the BOT-2 SF; therefore, data from all participants (*n* = 10) were included in the data analyses. The best score for each trial of the assessments was used in the analysis. Data were visually inspected for normality. Since the data did not appear normal for some of the variables, both parametric and non-parametric analyses were conducted. The results for both analyses were the same, so parametric data were presented for ease of interpretation and discussion. To determine the test–retest reliability of the BOT-2 SF and its subtests as well as the standing long jump, intraclass correlation coefficients (ICC) and 95% confidence intervals (CIs) were calculated. A two-way mixed model approach, mean rating (*k* = 1) with absolute agreement was used. The single measures ICC and 95% CIs are reported. Reliability was classified based on Shrout and Fleiss (1979) standards in which an ICC < 0.40 was considered to have ‘‘poor’’ reliability, 0.40 to 0.75 was considered to have “fair to good’’ reliability, and >0.75 was considered to have ‘‘excellent’’ reliability [38]. Standard error of measurement (SEM) was calculated using the formula (SEM = SD * √(1-ICC)). These statistics provide an indication of the error associated with the specific assessments.

Significance was set at an alpha level of *p* < 0.05. Analyses were conducted to determine effect sizes and estimated sample sizes necessary to detect the differences that were found in the current study. Additional participants needed to detect differences ranged from 28 to 3000 participants. Only one variable (transferring pennies) would benefit from an additional five participants (*n* = 15). Therefore, it was determined that recruiting additional participants would not significantly impact the findings from this study.

## 3. Results

Ten individuals participated in the study. Demographic information for the participants is presented in Table 2. The BOT-2 SF overall scores and percentile rankings test–retest reliability was considered excellent (ICC = 0.86, 95% CI: 0.54 to 0.96, *p* < 0.001, SEM = 1.46; percentile rankings: ICC = 0.84, 95% CI 0.50 to 0.96, *p* = 0.001, SEM = 0.64). The test–retest reliability results are presented for each of the subtests in Table 3. Five of the subtest assessments (folding paper, copying a square, standing on a balance beam, one-legged stationary hops, and knee push-ups) were classified as “poor” reliability. Six assessments were considered to have “fair to good” reliability (copying a star, transferring pennies, jumping in place, tapping finger and feet, dropping and catching a ball, and dribbling a ball) and the remaining assessments (drawing a line through crooked paths, walking forward on a line, sit-ups, and standing long jump) had excellent reliability (Table 4). With the exception of *running speed and agility,* all of the BOT-2 SF subtests had at least one assessment with fair to good or excellent reliability categories. The SEM for the subtest assessments ranged from 0.35 to 6.04; specific values for the individual assessments are included in Table 3.

## 4. Discussion

Overall, the BOT-2 SF appeared to be a reliable test for assessing the motor skill proficiency of youth with Down syndrome, indicating its use in research settings. To the authors’ knowledge, no studies have investigated the test–retest reliability of the BOT-2 SF in youth with Down syndrome. However, Wuang and Su (2009) explored the reliability of the BOT-2 CF in youth (ages 4 to 12 years old) with various causes of intellectual disabilities. In this investigation, the reliability ranged from 0.88 to 0.99. Additionally, the total score had an ICC of 0.99, indicating excellent reliability. The researchers concluded that the BOT-2 CF was a reliable test to assess the motor proficiency in youth with intellectual disabilities [34]. Conversely, Lam and colleagues (2018) explored the test–retest reliability of the BOT-2 CF in youth with Prader–Willi syndrome (ages 8 to 12 years old). The ICCs ranged from 0.47 to 0.97 across all tests. The total score had an ICC of 0.81, indicating excellent reliability. The researchers concluded that based on the reliability scores that the BOT-2 CF is appropriate to assess the motor skills of youth with Prader–Willi syndrome [31]. However, the BOT-2 CF was below the recommended threshold (ICC ≥ 0.90) presented by Nunnally and Bernstein (1994) and thus not appropriate for use within the clinical setting [39]. The test–retest reliability of the current investigation (overall ICC = 0.85) was slightly higher than that of Lam et al. (2018); however, the total score ICC was still lower than the 0.90 threshold for clinical use. Still, these findings suggest that this tool can effectively be used in a research setting (>0.80) as defined by Nunnally and Bernstein [39]. Direct comparison of all of these studies is not possible, since different populations and different versions of the assessments were utilized. It is suggested that future investigations should continue to explore the reliability and feasibility of the BOT-2 SF for individuals with Down syndrome and other populations with intellectual and developmental disabilities.

The majority of subtest assessments (67%) in the current study had ICCs greater than 0.40, indicating that the reliability was “fair to good”, with four of the assessments falling in the “excellent” category of reliability. The results in Table 3 indicate that there was variability throughout the BOT-2 SF, as well as within the different subtests. Some of the subtest assessments had low ICCs, which may have been a result of the participants being unaccustomed to these activities or that the activities were more difficult to accomplish. For example, for the *balance* subtest, the ICC for walking on a line (0.76) was much higher than the task of standing on a balance beam on one leg (0.10). The second task is much more demanding in terms of balance and skill level, whereas walking on line is a similar task to the type of walking that the participants do on a regular basis. The low ICCs may simply be a function of the lack of exposure or practice of the participants in those particular tasks. The findings from this study indicate that even though there is variability amongst the different subtest assessments, the BOT-2 SF showed excellent test–retest reliability and may be used in youth with Down syndrome in order to assess motor proficiency.

The results of this investigation suggest that the BOT-2 SF is a reliable test that can be used to assess overall motor proficiency in youth with Down syndrome. This study has several strengths, including the use of a single researcher to collect all of the data to ensure consistency of the assessments. Additionally, all participants attended both visits and completed all assessments at each visit to provide a complete dataset for analysis. The current investigation is not without limitations. Data were not collected on socio-economic status or sports that the participants practiced. These factors may influence motor proficiency levels. The average age of the participants was 17.7 years old, which may have influenced motor skills, as older youths may have performed better on the BOT-2 SF when compared to younger individuals. Additionally, the level of intelligence of the participants was not evaluated in this study, and therefore, the authors cannot determine if this factor impacted the assessment results. This is important, as cognition and language development have been reported to be strongly associated with both fine and gross motor skills, and this association may be strong in those with intellectual disabilities [40]. Thus, intelligence level may impact the ability to interpret the instructions and perform the assessments. An important limitation to this study that should be noted is the small sample size. The prevalence of youth with Down syndrome in the United States is low (≈0.001% of the population [41]) and is similar to worldwide estimates according to the World Health Organization, leading to difficulty recruiting participants. However, power and sample analyses determined that additional participants (e.g., 5 participants) would potentially only impact the findings related to one of the BOT-2 SF variables (transferring pennies). Despite these limitations, the results of the current investigation demonstrate that this assessment is a reliable measurement tool for this population. All of the youth enrolled in the current study were highly functioning individuals, who appeared to be able to understand and follow the directions for the BOT-2 SF. Therefore, these findings may not be generalizable to all individuals with Down syndrome. Additionally, since normative data for this population have not been established, future research should develop standards specific to this population to better classify motor deficiencies.

Due to the potential impact that motor skill proficiency has on physical activity levels and overall health of individuals, including persons with Down syndrome, it is paramount for clinicians and researchers to determine if these tests are reliable. The BOT-2 SF, which is often used in a variety of settings, may be a feasible assessment tool. The results of this investigation suggest that the BOT-2 SF along with a standing long jump can be used in the research setting to assess the motor proficiency of youth with Down syndrome.

## Figures and Tables

**Table 1:** Assessments Included in Each Subtest of the Bruininks–Oseretsky Test of Motor Proficiency–2 Short Form (BOT-2 SF).

Subtest of Motor Proficiency	Subtest Assessment	Assessment Criteria *
**Fine Motor Precision**	Drawing lines through crooked paths Folding paper	Must draw inside of the maze lines, points deducted for drawing outside of the lines or touching the lines Folds must align with fold lines on the paper, points are deducted for not aligning with the fold lines
**Fine Motor Integration**	Copying a square Copying a star	Specific criteria regarding the basic shape, lines closures, edges, orientation, and overall size for the square and star
**Manual Dexterity**	Transferring pennies	Pick up penny with left hand, transfer penny to right hand and then to box. The number of pennies transferred in 15 s is recorded. Trial does not count if participant deviates from proper form.
**Body Coordination**	Jumping in place (same sides synchronized) Tapping feet and fingers (same sides synchronized)	Jumps must have arm and leg on the same side synchronized, count up to 5 correct jumps. Jumps with improper form, such as arm and leg are not synchronized do not count. Tapping feet and fingers on same side up to 10 correct taps. Taps do not count if movement is not continuous or the participant fails to tap the same side.
**Balance**	Walking forward on a line Standing on one leg on a balance beam with eyes open	Count number of correct steps on a straight line up to 6 steps. Steps do not count if the participant is off the line or hands not on hips. Count the number of seconds the participant stands using proper form. Timer is stopped if the participant fails to keep leg raised or falls off beam.
**Running Speed and Agility**	One legged stationary hops	Count number of correct hops in 15 s. Hops not counted if the raised foot touches the floor or if the participant stumbles off of starting point.
**Upper-Limb Coordination**	Dropping and catching a ball with both hands Dribbling a ball with alternating hands	Count number of catches up to 5. Catch does not count if the participant misses the ball or traps the ball to the body. Count number of correct dribbles up to 10. Dribble does not count if the participant does not alternate hands or if there is more than one bounce between dribbles.
**Strength**	Push-ups (full or knee) Sit-ups	Count of the number of correct push-ups in 30 s. Push-ups do not count if hips are lifted or if the arms do not reach 90 degrees. Count the number of correct sit-ups in 30 s. Sit-ups do not count if the participant pushes up with elbows or pulls body up using clothing.

* Full criteria for each subtest can be found in the BOT-2 manual [30].

**Table 2:** Descriptive characteristics (*n* = 10; mean + SD; range).

Characteristics	
**Age**	17.7 ± 3.0 (13.1–20.7)
**Mass (kg)**	58.9 ± 16.5 (37.8–84.5)
**Height (cm)**	152.0 ± 11.7 (123.5–167.3)
**BMI (kg/m^2^)**	25.2 ± 5.5 (17.7–35.3)
**Sex**	
Male	50%
Female	50%
**Ethnicity**	
Hispanic	0%
Non-Hispanic	100%
**Race**	
White	90%
Two or more races	10%

**Table 3:** BOT-2 SF subtest scores, intraclass correlations (95% CI), and standard error of the measurement (SEM).

BOT-2 SF Subtests	Trial 1 Mean ± SD; (Range)	Trial 2 Mean ± SD; (Range)	ICC (95% CI)	SEM
***Fine Motor Precision***				
Drawling Lines through Paths—Crooked (No. of errors)	2.4 ± 2.2; (0–6)	2.5 ± 2.2; (0–6)	0.89 (0.60–0.97)	0.73
Folding Paper (No. of errors)	1.5 ± 1.4; (0–5)	1.6 ± 1.8; (0–5)	0.31 (−0.44–0.78)	1.16
***Fine Motor Integration***				
Copying a Square (No. of errors)	4.8 ± 0.4; (4–5)	4.1 ± 1.3; (1–5)	0.12 (−0.37–0.64)	0.38
Copying a Star (No. of errors)	2.5 ± 2.2; (0–5)	2.2 ± 2.0; (0–5)	0.48 (−0.21–0.84)	1.44
***Manual Dexterity***				
Transferring Pennies (No. of pennies)	2.3 ± 1.3; (1–5)	3.0 ± 1.2; (1–5)	0.69 (0.08–0.92)	0.67
***Bilateral Coordination***				
Jumping in Place—Same Sides Synchronized (No. of jumps)	2.4 ± 1.1; (0–3)	2.7 ± 0.5; (2–3)	0.50 (−0.10–0.85)	0.35
Tapping Feet and Fingers—Same Sides Synchronized (No. of taps)	3.5 ± 1.3; (0–4)	3.0 ± 1.6; (0–4)	0.61 (0.06–0.88)	0.81
***Balance***				
Walking Forward on a Line (No. of steps)	2.7 ± 0.9; (2–4)	2.9 ± 0.9; (2–4)	0.76 (0.32–0.93)	0.44
Standing on One Leg on a Balance Beam—Eyes Open (sec)	1.2 ± 0.9; (0–3)	1.2 ± 0.8; (0–2)	0.10 (−0.64-0.68)	0.76
***Running Speed and Agility***				
One-Legged Stationary Hops (No. of hops)	3.2 ± 1.8; (0–6)	4.2 ± 1.8; (1–7)	0.30 (−0.27–0.75)	1.51
***Upper-Limb Coordination***				
Dropping and Catching a Ball—Both Hands (No. of catches)	3.7 ± 1.6; (0–5)	4.2 ± 0.8; (3–5)	0.44 (−0.18–0.82)	0.60
Dribbling a Ball—Alternating Hands (No. of dribbles)	4.6 ± 1.8; (1–7)	4.1 ± 2.1; (0–6)	0.61 (0.02–0.88)	1.12
***Strength***				
Knee Push-Ups (No.)	2.5 ± 2.4; (0–6)	3.8 ± 2.3; (0–7)	0.26 (−0.31–0.73)	1.98
Sit-Ups (No.)	3.0 ± 1.7; (0–6)	2.9 ± 1.4; (1–5)	0.86 (0.53–0.96)	0.52
Standing Long Jump (cm)	30.3 ± 15.6; (10–59)	32.0 ± 17.4; (14–75)	0.85 (0.50–0.96)	6.04

**Table 4:** Classification of the test–retest reliability (1CC; 95% CI) for the BOT-2 SF subtest and overall assessment scores.

Classification of Subtest ICCs	Subtest Categories	No. of Subtests (%)
***Excellent reliability (≥0.75*** *)*		***4/15 (27%)***
• Drawling Lines through Paths—Crooked (No. of errors)	Fine Motor Precision	
• Walking Forward on a Line (No. of steps)	Balance	
• Sit-Ups (No.)	Strength	
• Standing Long Jump (cm)	Strength	
***Fair to good reliability (≥0.40–<0.75)***		***6/15 (40%)***
• Copying a Star (No. of errors)	Fine Motor Integration	
• Transferring Pennies (No. of pennies)	Manual Dexterity	
• Jumping in Place—Same Sides Synchronized (No. of jumps)	Bilateral Coordination	
• Tapping Feet and Fingers—Same Sides Synchronized (No. of taps)	Bilateral Coordination	
• Dropping and Catching a Ball—Both Hands (No. of catches)	Upper Limb Coordination	
• Dribbling a Ball—Alternating Hands (No. of dribbles)	Upper Limb Coordination	
***Poor reliability (<0.40*** *)*		***5/15 (33%)***
• Folding Paper (No. of errors)	Fine Motor Precision	
• Copying a Square (No. of errors)	Fine Motor Integration	
• Standing on One Leg on a Balance Beam—Eyes Open (sec)	Balance	
• One-Legged Stationary Hops (No. of hops)	Running Speed and Agility	
• Knee Push-Ups (No.)	Strength	
***Excellent reliability (*** ***≥0.75*** *)*		***2/2 (100%)***
• Overall Score	Overall Assessment	
• Percentile Ranking	Overall Assessment	

## Data Availability

The data presented in this study are available on request from the corresponding author. The data are not publicly available due to privacy.
